# Radiation Impairs Perineural Invasion by Modulating the Nerve Microenvironment

**DOI:** 10.1371/journal.pone.0039925

**Published:** 2012-06-29

**Authors:** Richard L. Bakst, Nancy Lee, Shuangba He, Natalya Chernichenko, Chun-Hao Chen, Gary Linkov, H. Carl Le, Jason Koutcher, Efsevia Vakiani, Richard J. Wong

**Affiliations:** 1 Department of Radiation Oncology, Memorial Sloan-Kettering Cancer Center, New York, New York, United States of America; 2 Department of Surgery, Memorial Sloan-Kettering Cancer Center, New York, New York, United States of America; 3 Department of Medical Physics, Memorial Sloan-Kettering Cancer Center, New York, New York, United States of America; 4 Department of Medicine, Memorial Sloan-Kettering Cancer Center, New York, New York, United States of America; 5 Department of Pathology, Memorial Sloan-Kettering Cancer Center, New York, New York, United States of America; NIH, United States of America

## Abstract

**Purpose:**

Perineural invasion (PNI) by cancer cells is an ominous clinical event that is associated with increased local recurrence and poor prognosis. Although radiation therapy (RT) may be delivered along the course of an invaded nerve, the mechanisms through which radiation may potentially control PNI remain undefined.

**Experimental Design:**

An *in vitro* co-culture system of dorsal root ganglia (DRG) and pancreatic cancer cells was used as a model of PNI. An *in vivo* murine sciatic nerve model was used to study how RT to nerve or cancer affects nerve invasion by cancer.

**Results:**

Cancer cell invasion of the DRG was partially dependent on DRG secretion of glial-derived neurotrophic factor (GDNF). A single 4 Gy dose of radiation to the DRG alone, cultured with non-radiated cancer cells, significantly inhibited PNI and was associated with decreased GDNF secretion but intact DRG viability. Radiation of cancer cells alone, co-cultured with non-radiated nerves, inhibited PNI through predominantly compromised cancer cell viability. In a murine model of PNI, a single 8 Gy dose of radiation to the sciatic nerve prior to implantation of non-radiated cancer cells resulted in decreased GDNF expression, decreased PNI by imaging and histology, and preservation of sciatic nerve motor function.

**Conclusions:**

Radiation may impair PNI through not only direct effects on cancer cell viability, but also an independent interruption of paracrine mechanisms underlying PNI. RT modulation of the nerve microenvironment may decrease PNI, and hold significant therapeutic implications for RT dosing and field design for patients with cancers exhibiting PNI.

## Introduction

Perineural invasion (PNI) has been broadly defined as tumor cell invasion, in, around and through nerves [1]. PNI is a frequent clinical and pathological finding in head and neck, pancreatic, prostate, and other cancers [2], and has been shown to be a marker of poor outcome, with increased locoregional recurrence rates and decreased survival [3]–[5]. Support for the clinical application of radiation therapy (RT) in the treatment of cancers with PNI is derived primarily from limited retrospective series demonstrating improved local control rates following radiation of neurotrophic cancers [6]–[9]. However, we currently lack a biological understanding of how radiation treatment of PNI translates into improved disease control. In current head and neck cancer clinical practice, full therapeutic doses of radiation may be applied to cranial nerves exhibiting PNI along the entire course of the nerve to the skull base. A mechanistic justification for such clinical practice, however, is lacking.

Early theories on PNI suggested that its pathogenesis was centered on neural sheaths serving as a low-resistance conduit for tumor cell growth, or through lymphatic channels [10], [11]. More recent studies have suggested that PNI involves signaling amongst tumor, nerve, and stromal cells through paracrine mechanisms [12], [13]. Neurotrophic and axonal guidance molecules have potent effects on axonal growth, and may be upregulated in cancers with a predilection for nerve invasion [14]–[16]. Neurotrophic factors secreted in a gradient along nerves [17] may play a pivotal role in PNI pathogenesis. Our group recently demonstrated that nerve secretion of glial-derived growth factor (GDNF) activates RET-receptor mediated cancer cell chemotaxis, guiding directional cell migration towards and along nerves [18].

In the current study, we examine how RT may mechanistically inhibit PNI in the context of this new paradigm, emphasizing the importance of considering the dynamic interactions occurring between cancer cells and the nerve microenvironment. We hypothesized that RT may impair PNI through both direct effects on cancer cell viability, and also by potentially altering the nerve microenvironment through a disruption of neurotrophic gradients. This approach was based on powerful *in vitro* and *in vivo* models of PNI that permit the study of interactions between cancer cells and nerve.

## Materials and Methods

### Ethics statement

All mouse studies were performed in accordance with institutional protocol guidelines at Memorial Sloan-Kettering Cancer Center (MSKCC). Mice were maintained according to NIH Animal Care guidelines, under protocols approved by the MSKCC Institutional Animal Care Committee describing experiments specific to this study (protocol number 05-04-006). Studies on human tissue samples were approved by the MSKCC Institutional Review Board. Written informed consent was received from all participants.

**Figure 1:**
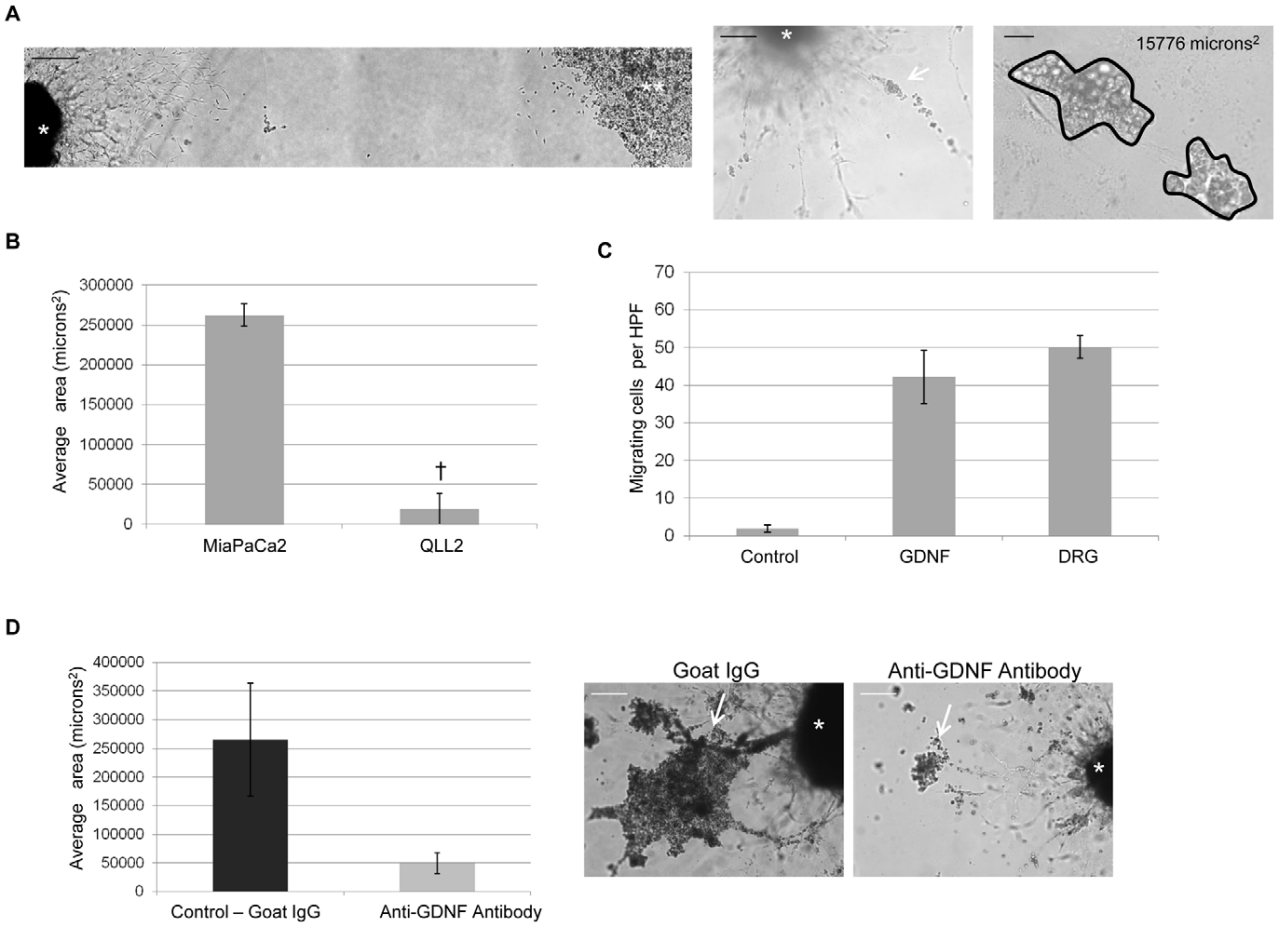
GDNF-mediated cancer cell migration and a DRG co-culture model of PNI. A. MiaPaCa2 pancreatic cancer cells were (**) grown in a culture insert adjacent to a DRG (*) in Matrigel. Bar, 500 µm. DRG becomes invaded by MiaPaCa2 (arrow) which track along a neurite. Bar, 250 µm. Area of invasion was calculated using Metamorph computer software, 8 days after insert removal. Bar, 50 µm. B. MiaPaCa2 reliably invades DRG, in contrast to the squamous cell carcinoma cell line QLL2 († p<0.05, t-test). C. MiaPaCa2 migrate towards both GDNF and DRG in Boyden chamber assays. D. DRG treated with anti-GDNF antibodies demonstrated decreased area of invasion by MiaPaCa2 (p = 0.15, t-test). Photomicrographs show representative decreased area of invasion (arrow) at day 8 with anti-GDNF antibody treatment in comparison to control. Bar, 250 µm.

### Cell lines, reagents, antibodies

Human pancreatic adenocarcinoma (MiaPaCa2) and head and neck squamous cell carcinoma (QLL2) cell lines were used. MiaPaCa2 was purchased from the American Type Culture Collection (Manassas, VA). QLL2 was derived from a patient at Memorial Sloan-Kettering Cancer Center (MSKCC) [19]. Cells were grown *in vitro* in Dulbecco's modified Eagle medium (DMEM) containing 10% fetal calf serum (FCS), penicillin, and streptomycin, and incubated in 5% CO2-humidified incubator at 37°C. GDNF was obtained from EMD Chemicals (Rockland, MA). Anti-GDNF antibody (5 µg/mL) was obtained from Abcam (Cambridge, MA).

**Figure 2:**
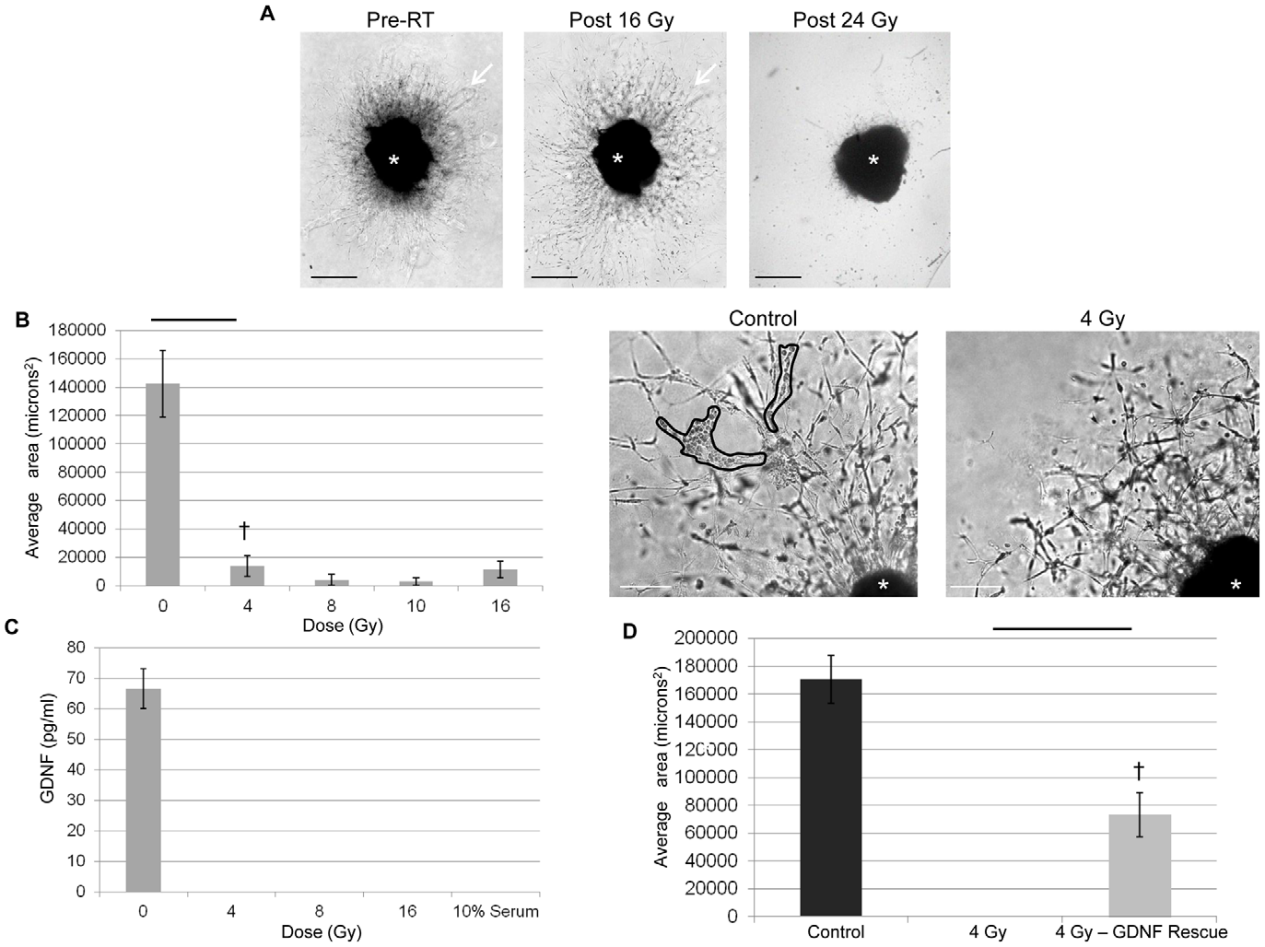
Radiation to DRG alone impairs PNI *in vitro*. A. DRG (*) remained viable without morphological change after a single dose of 16 Gy; however, a single dose of 24 Gy results in a loss of neurites (arrows) consistent with DRG death. Bar, 500 µm. B. DRG co-culture model of PNI was performed. Non-radiated MiaPaCa2 cells were grown in an insert adjacent to a DRG exposed to a single dose of radiation (4–16 Gy) in Matrigel. The insert was removed, and 8 days later the area of invasion was calculated using Metamorph computer software. Low doses of radiation to the DRG significantly suppressed invasion by untreated cancer cells († p<0.05, t-test). A photomicrograph of a DRG (*) 8 days after exposure to 4 Gy shows no PNI, in contrast to control, non-radiated DRG (area of invasion outlined). Bar, 250 µm. C. GDNF ELISA was performed on conditioned media from DRG 8 days after a single radiation exposure. Single doses of radiation as low as 4 Gy suppressed GDNF to levels below the limit of detection (31 pg/mL). D. GDNF supplementation partially rescues invasion following RT. DRG were grown in Matrigel supplemented with GDNF (150 ng) and then radiated to 4 Gy. GDNF was added (60 ng) to the DRG every other day, which partially restored the mean area of invasion (p<0.05, t-test).

### Colony forming assays

MiaPaCa2 were irradiated in single-fraction doses between 2 and 10 Gy. Cells ranging from 1×10^2^ to 5×10^3^ were plated, and colonies were stained with cresyl violet and counted 8 days later. Results are expressed as surviving fraction accounting for plating efficiency. All experiments were performed in triplicate.

**Figure 3:**
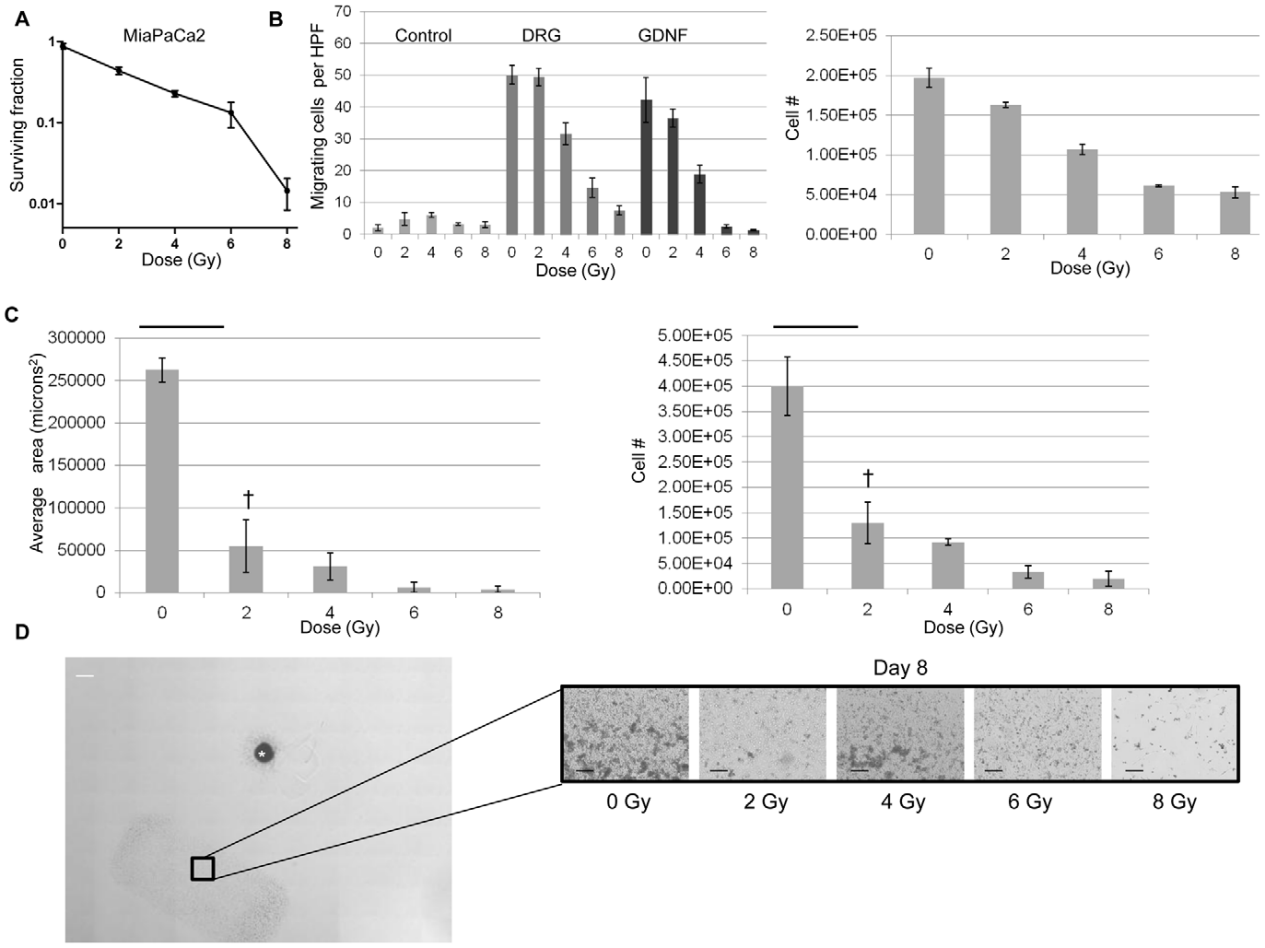
Radiation of MiaPaCa2 cells impairs migration and PNI predominantly through compromised cellular viability. A. Colony-forming assays of MiaPaCa2 cells show susceptibility to single-fraction radiation doses, with logarithmically diminishing surviving fractions up through 8 Gy. B. MiaPaCa2 cells were radiated at varying single fraction doses, 24 hours later serum starved overnight, and then added (2×10^5^) to the upper chamber of migration assays. The lower chamber contained control media, DRG, or GDNF (100 ng/mL). Radiation reduced the number of migrating cells towards DRG or GDNF in a dose-dependent fashion. MiaPaCa2 were radiated and simultaneously plated with migration assays, under identical conditions, and counted after 24 hours. Dose-dependent decreases in cancer cell number, attributable to radiation induced cell death, correlated with results from migration assay. C. Single fraction doses of radiation (2–8 Gy) were applied to MiaPaCa2 cells prior to their addition to the DRG co-culture model of PNI. Radiation doses as low as 2 Gy significantly decreased the area of invasion († p<0.05, t-test). MiaPaCa2 were radiated and simultaneously plated with DRG co-culture assays, under identical conditions, and counted after 8 days. Dose-dependent decreases in cancer cell number, attributable to radiation induced compromised cell viability, correlated with decreases in area of invasion. D. MiaPaCa2 colonies adjacent to the DRG (*) at day 8 show diminished viability with increasing radiation dose, consistent with cell numbers quantified in Fig. 3C. White bar, 1000 µm, black bar, 250 µm.

### Boyden chamber migration assays

Polyethylene terephthalate 8.0 µm pore inserts (BD Biosciences, Bedford, MA) were used in 24-well plates. Cancer cells were incubated in 0.1% FCS media overnight, and 2×10^5^ cells were added to each insert in 0.5 mL of media with 0.1% FCS. Dorsal root ganglia (DRG) from newborn male Balb/c mice were isolated as previously described [18], [20]. Below the inserts, 0.7 mL of 0.1% FCS media was added to each of the wells with GDNF (100 ng/mL), DRG, or DRG radiated by single doses up to 16 Gy. The inserts were removed after 24 hours, and non-migrating cells wiped off with a cotton swab. The migrating cells on the undersurface of the membrane were fixed in 100% alcohol and stained with 1% methylene blue in 1% borax. Membranes were excised and mounted on glass slides. Cells were counted at five high-power fields at predetermined areas of the membrane.

**Figure 4:**
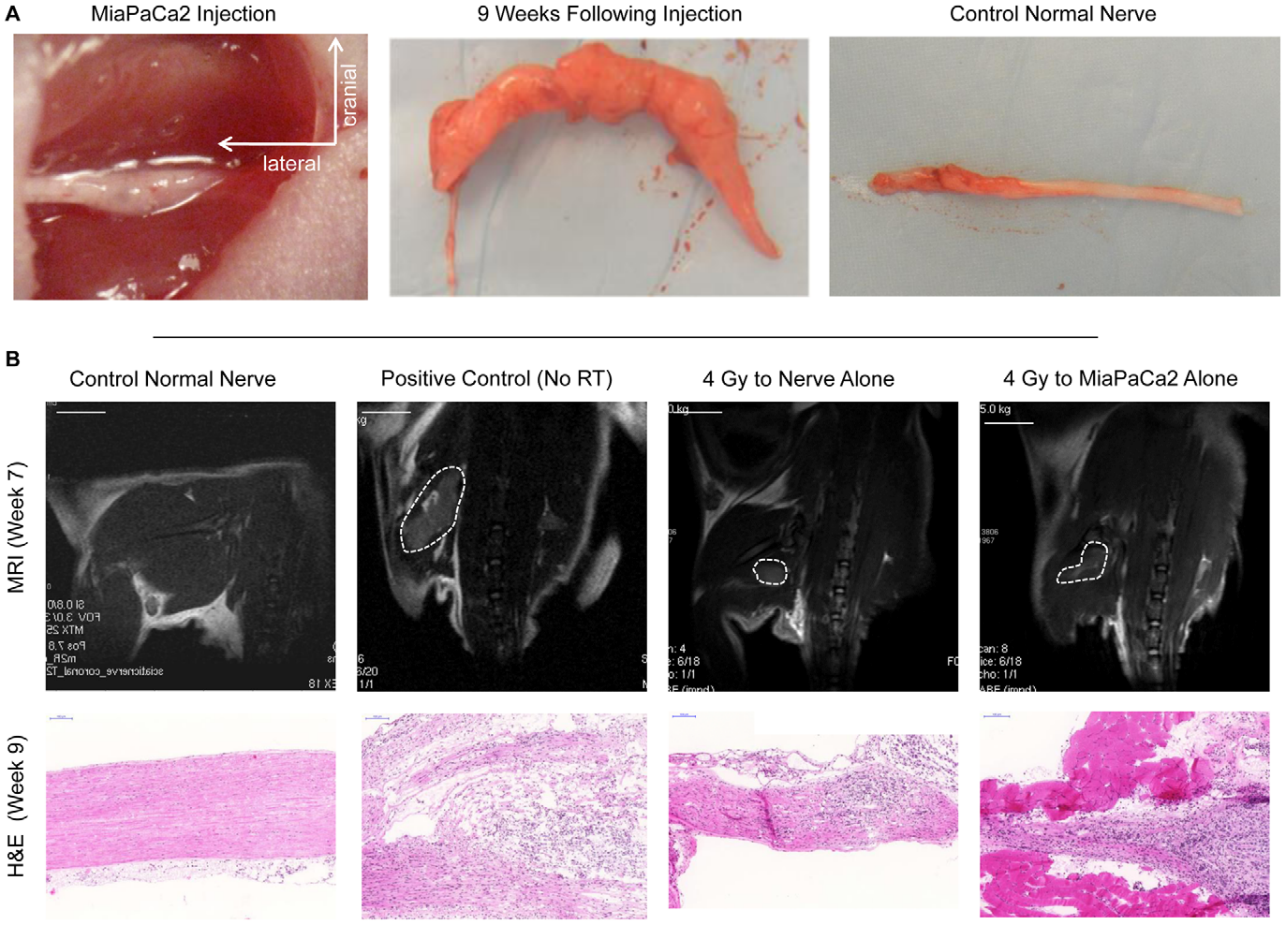
PNI *in vivo* is impaired following radiation. A. Untreated MiaPaCa2 cells were implanted into the distal sciatic nerve of mice under anesthesia. Nine weeks later, there is gross thickening along the course of the nerve, consistent with PNI in comparison to a normal sciatic nerve. B. MRI (T2 weighted image) and hematoxylin and eosin (H&E) staining of a mouse injected with PBS into the sciatic nerve respectively show a thin, barely visible, sciatic nerve (bar, 1.0 cm) with normal nerve histology (bar, 100 µm). MRI and H&E of a non-radiated mouse after injection with non-radiated MiaPaCa2 cells respectively demonstrate a thick and enhancing right sciatic nerve extending toward the spine cord and extensive invasion of the nerve. MRI and H&E of a mouse radiated to 4 Gy to the sciatic nerve only, respectively show decreased nerve thickness and PNI, as compared with control. MRI and H&E after injection of a non-radiated mouse with MiaPaCa2 cells radiated to 4 Gy respectively show decreased nerve thickness and PNI as compared with control.

### *In vitro* co-culture model of nerve invasion

Excised DRG were implanted in growth factor–depleted Matrigel matrix (BD Biosciences Bedford, MA) adjacent to a cell culture insert containing a divider (Ibidi, Munchen, Germany). At day 5 after DRG explantation, 1×10^4^ cancer cells were added to the insert and the divider was removed 24 hours later to permit a controlled starting point for the onset of interactions. The area of invasion into the DRG was calculated 8 days following culture insert removal using software (MetaMorph 7.7.4; Molecular Devices, Sunnyvale, CA) to outline the area invaded by cancer cells. Images were acquired on an Axiovert 200M microscope (Carl Zeiss, Oberkochen, Germany) using a Photometrics Coolsnap ES camera (Photometrics, Tucson, AZ).

**Figure 5:**
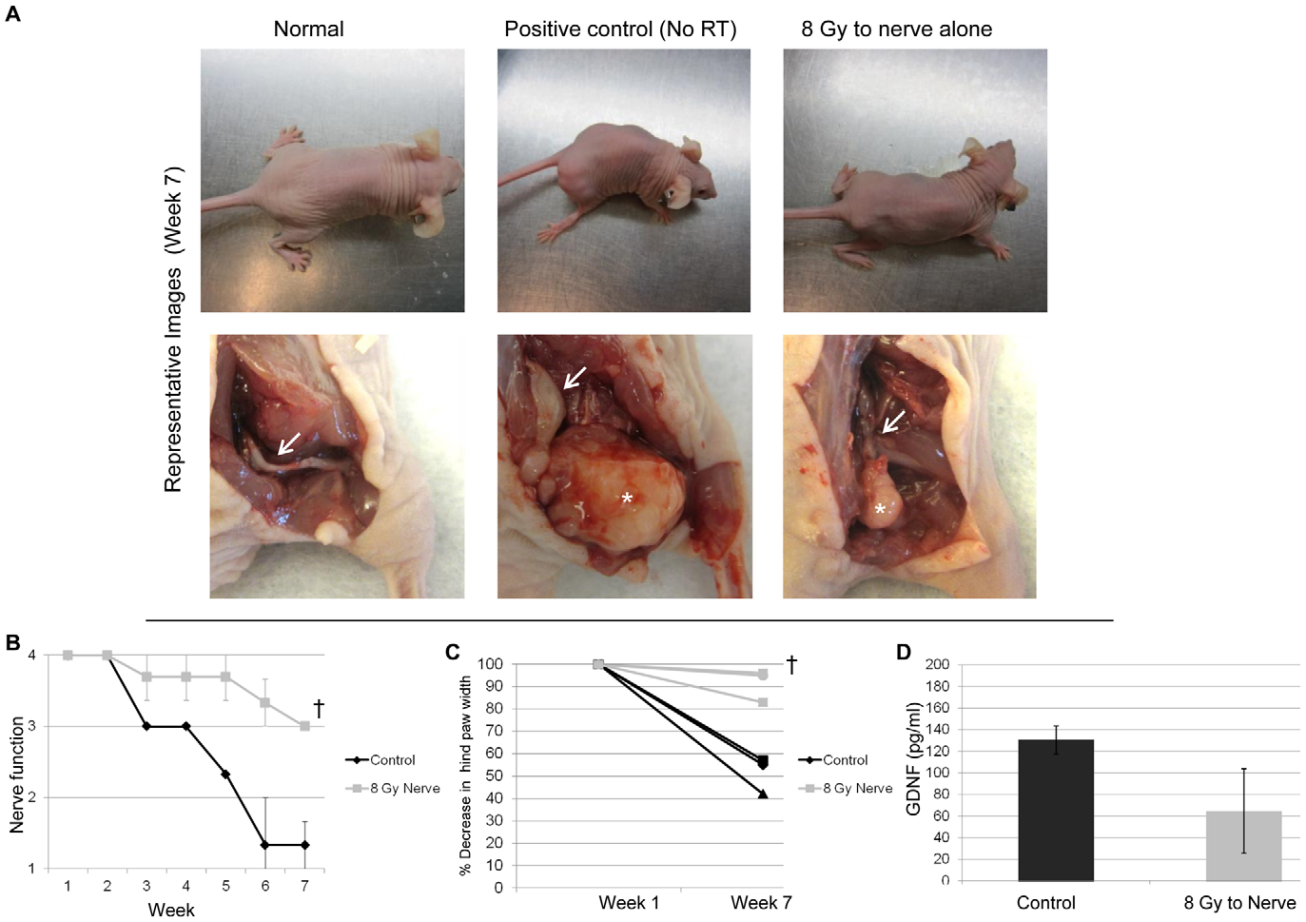
Radiation to the sciatic nerve alone impairs PNI and preserves nerve function *in vivo*. A. Representative images of a mouse injected with PBS into the right sciatic nerve demonstrate normal bilateral hind limb function and a normal sciatic nerve *in situ*. Representative images of a mouse 7 weeks after injection of MiaPaCa2 into the right sciatic nerve demonstrate paralysis of the right hind limb and gross tumor (*) with PNI and sciatic nerve thickening (arrow) *in situ*. Representative images of a mouse that received 8 Gy of radiation to the right sciatic nerve which was then injected with non-radiated MiaPaCa2 cells. Seven weeks later, right hind limb function remains intact and a smaller gross tumor (*) with no sciatic nerve thickening (arrow) *in situ*. B. Radiation to the sciatic nerve, prior to cancer injection, preserves hind limb function. Mean right sciatic nerve function score of mice receiving 0 (control) or 8 Gy to the right sciatic nerve prior to injection of non-radiated MiaPaCa2 cells († p<0.05, t-test). A nerve score of four indicates normal hind limb function, and a score of one indicates complete paralysis. C. The sciatic nerve index (hindpaw span) was used as a measure of sciatic nerve function 7 weeks after MiaPaCa2 injection in the right sciatic nerve of mice receiving 0 (control) or 8 Gy of radiation to the right sciatic nerve prior to injection of non-radiated MiaPaCa2 cells. Radiation to sciatic nerves preserves nerve function in comparison to non-radiated nerves († p<0.05, t-test). D. GDNF production in sciatic nerves decreases 3 weeks after a single radiation dose (8 Gy) as compared with controls (0 Gy) (p = 0.13, t-test).

For studies blocking GDNF, anti-GDNF antibody (5 µG/mL) was added to media every other day, with goat immunoglobulin G used as control. For studies using RT, single fraction doses of radiation were administered to either cancer cells or DRG using a Shepard Mark-1 Cs-137 irradiator. Radiated cancer cells were added to inserts twenty-four hours after radiation. Non-radiated cancer cells were added to radiated DRG 24 hours after radiation. For GDNF rescue experiments, DRG were explanted and grown in Matrigel with GDNF (150 ng) for 5 days, and then radiated to 4 Gy. After cancer cells were added to the insert, GDNF (60 ng) was added into the Matrigel drop every other day. Phosphate-buffered saline (PBS) added to the non-radiated DRG served as controls.

**Figure 6:**
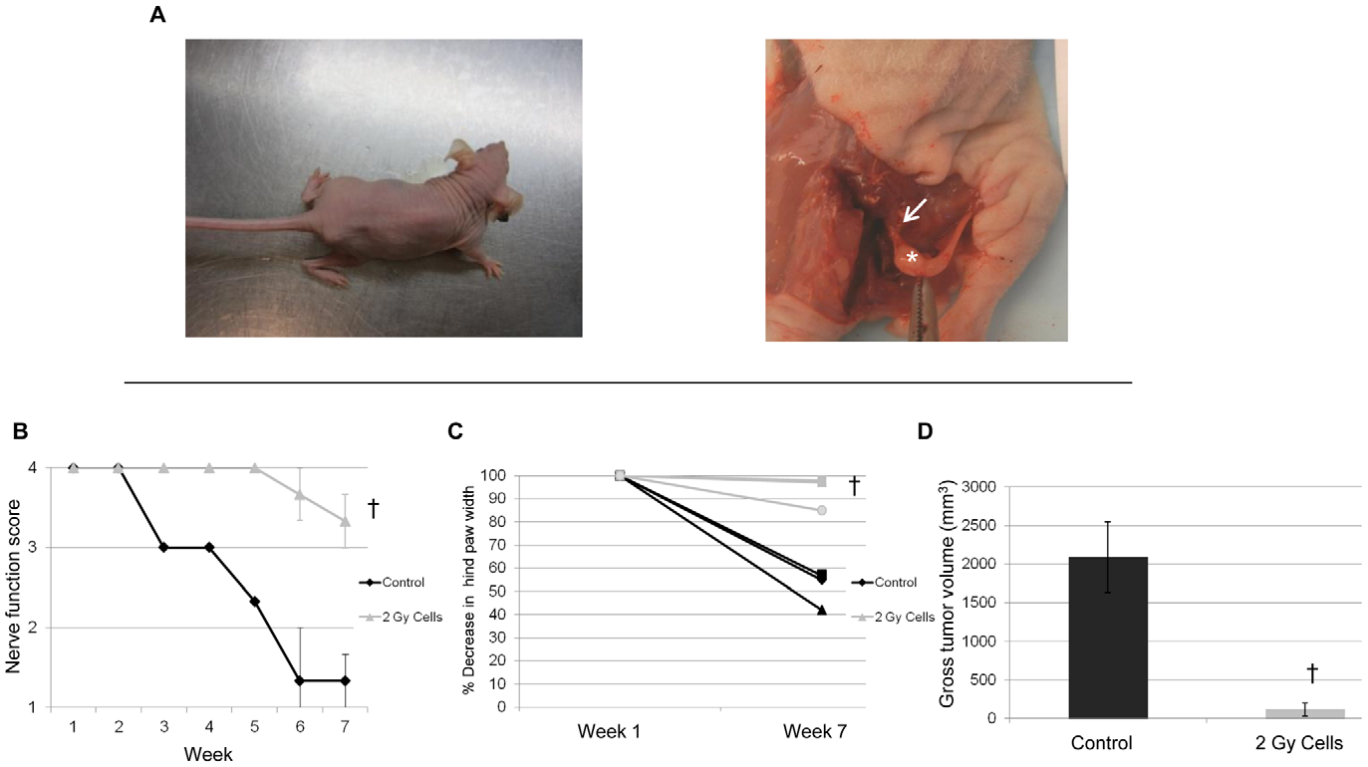
Radiation to cancer cells alone diminishes PNI *in vivo*. A. Representative images of a non-radiated mouse 7 weeks after sciatic nerve injection with radiated (2 Gy) MiaPaCa2 cells demonstrate intact right hind limb function and diminished gross tumor (*) with normal sciatic nerve caliber (arrow) *in situ*, in contrast to non-radiated MiaPaCa2 cells (Fig. 5A). B. Radiation to cancer cells alone prevents sciatic nerve paralysis. Mean right sciatic nerve function scores were measured 7 weeks after mice underwent sciatic nerve injections with radiated (2 Gy) or control (0 Gy) MiaPaCa2 cells († p<0.05, t-test). C. The sciatic nerve index (hindpaw span) was used as a measure of sciatic nerve function 7 weeks after injection of radiated (2 Gy) or control (0 Gy) MiaPaCa2 cells into the right sciatic nerve. Mice injected with radiated cancer cells exhibit preserved neurological function as compared to mice receiving non-radiated control cancer cells († p<0.05, t-test). D. Mean gross tumor volumes assessed 7 weeks after sciatic nerve injections demonstrate that cancer cells receiving radiation (2 Gy) exhibited significantly smaller tumor volumes as compared with control (0 Gy) cancer cells († p<0.05, t-test).

### *In vivo* model of murine sciatic nerve invasion

Radiation was administered to selected nude athymic mice using an X-RAD 320 animal irradiator (Precision X-Ray, North Branford, CT). Mice were given ketamine (1.5 mg/mL) prior to radiation. Single-fraction doses up to 16 Gy were administered along the entire course of the sciatic nerve up to the spinal cord utilizing a custom lead shield (Precision X-Ray, North Branford, CT).

One week after radiation to the sciatic nerve, mice were anesthetized using isoflurane (5% for induction, 2% for maintenance), and their sciatic nerves were surgically exposed. The nerve was identified deep to the femerococcygeous and biceps femoris muscles. MiaPaCa2 cells were microscopically injected into the distal sciatic nerve under the epineurium. Microinjection of 3 µL of cell suspension at a concentration of 2×10^5^ cells/μL was performed using a custom-made Hamilton syringe.

**Figure 7:**
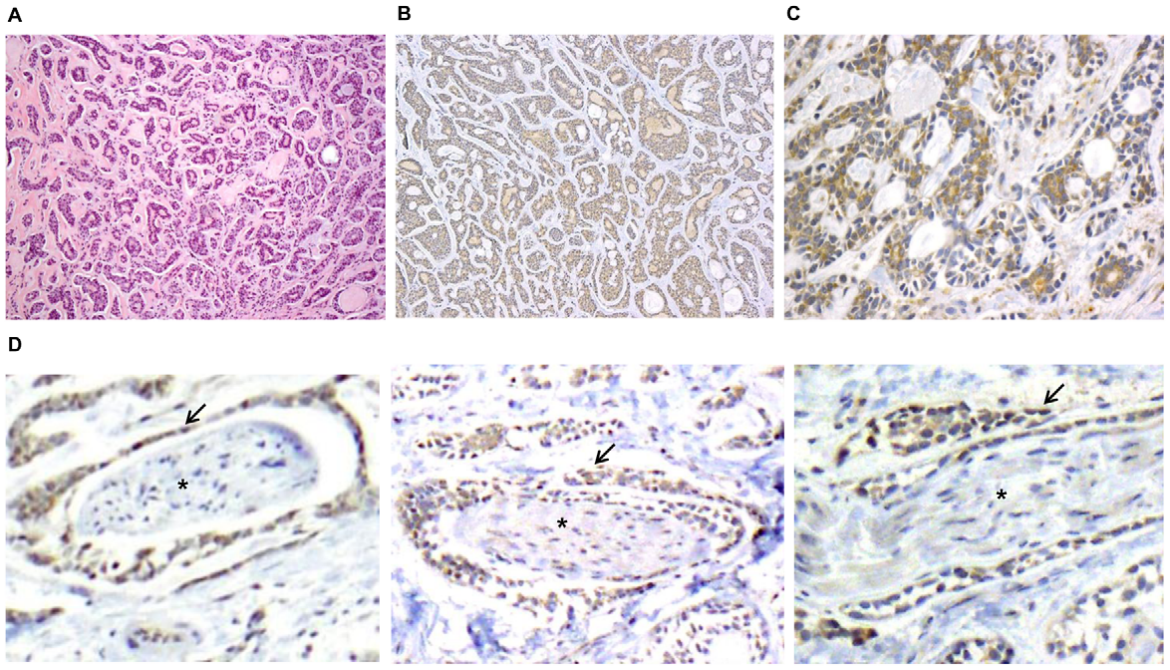
RET is expressed widely in human adenoid cystic carcinoma (ACC), which frequently exhibit PNI. A. Hematoxylin-eosin staining of an ACC specimen (10x) demonstrates well-formed ducts and nests of cells growing in hyalinized stroma in a mixed tubular and cribriform pattern. RET immunohistochemistry demonstrates widespread staining of ACC at both 10x (B.) and 20x (C.). D. RET immunohistochemistry at 40x demonstrates 3 cases of PNI, with RET positive expression by ACC cells (arrows) exhibiting PNI of small nerves (*).

Sciatic nerve function was measured weekly as described previously, utilizing gross behavior, sciatic neurological score (hind limb motor response to extension, 4 = normal and 1 = full paralysis) and sciatic nerve function index (hindpaw width) [18]. Sciatic nerve specimens were excised immediately following animal sacrifice, frozen in OCT, and cut into 8-µm-thick sections on glass slides. The slides were fixed and stained with hematoxylin and eosin and reviewed with a pathologist (E. Vakiani). Images were acquired with a Mirax slide scanner (20x/0.8 NA objective) utilizing Mirax scan software (Carl Zeiss, Oberkochen, Germany).

### GDNF production by DRG and sciatic nerves

Normal or radiated DRG were grown in 24-well plates for 13 days and the conditioned media removed and centrifuged. Supernatant was assayed for GDNF using an enzyme-linked immunosorbent assay (ELISA) kit (GDNF Emax® ImmunoAssay System, Promega, Madison, WI).

A single-fraction dose of 8 Gy was administered to the right sciatic nerve as described, with the non-radiated left nerve serving as control. Three weeks following radiation, mice were sacrificed and bilateral sciatic nerves were excised. Identical segments of nerve were removed. The nerves underwent homogenization with an electronic pestle grinder (Fisher Scientific, Pittsburgh, PA). Tissue lysates were centrifuged and analyzed by GDNF ELISA. Total protein was quantified for each nerve (Bio-Rad, Hercules, CA).

### MRI of sciatic nerves

Mice were subjected to magnetic resonance imaging (MRI), performed on a Bruker USR 4.7T 40-cm bore scanner (Bruker Biospin MRI GmbH, Ettlingen, Germany) equipped with a 400 mT/m 12-cm bore gradient, using a custom-designed active decoupled radiofrequency surface coil (Stark MRI Contrast Research, Erlangen, Germany). Mice were anesthetized with 1% to 1.5% Isoflurane (Baxter Healthcare Corp., Deerfield, IL) in oxygen and monitored with an animal physiological monitoring system (SA Instruments Inc., Stony Brook, NY). A scout fast spin echo scan in three orientations was acquired to localize the sciatic nerve, followed by an oblique-coronal T2-weighted fast spin-echo image acquired with: TR/TE 1.9 s and 40 ms, 117×186 µm in-plane resolution, 20 slices of 0.8 mm slice thickness and 16 averages. Contrast was injected via mouse tail vein 0.1 mmol/kg Gd-DTPA (gadolinium diethylenetriamine penta-acetic acid), Magnevist (Bayer Healthcare Pharmaceuticals Inc., Wayne, NJ). Oblique-coronal T1-weighted gradient-echo images were acquired continuously prior to and after injection. The acquisition parameters were TR 126 ms, TE 2.2 ms, 156×186 µm in-plane resolution, 12 slices with 0.8 mm slice thickness, 8 averages with a time resolution of 3 minutes.

### RET expression by human adenoid cystic carcinoma

Previously untreated patients with adenoid cystic carcinoma (ACC) were identified through a search of the MSKCC Pathology Tissue Bank. Primary tumor paraffin embedded tissue blocks were obtained. Hematoxylin and eosin stained slides were evaluated for the presence of nerve invasion by an experienced pathologist (E. Vakiani). Sections were cut at 5 µm thickness from paraffin blocks. Immunostaining for RET was performed with a Ventana automated staining system (Ventana Medical Systems, Tucson, AZ) and a polyclonal pig anti-human RET antibody (Abgent, San Diego, CA). A purified human medullary carcinoma cell line (TT) expressing RET was used as the positive control, and a purified fibroblast cell line pellet lacking RET was used as the negative control. Images of slides were captured on an Olympus BX 41 microscope using an Olympus DP20 camera (Olympus, Center Valley, PA).

### Statistical analyses

A student t-test was used for statistical analysis as appropriate. All P values were calculated using two-sided tests. Differences were considered statistically significant if P was less than .05. Error bars in the graphs represent 95% confidence intervals. Data from representative experiments are shown.

## Results

### *In vitro* model of nerve invasion by cancer

To investigate dynamic relationships between cancer and nerves, we developed a co-culture model using an insert divider as a temporary barrier that is removed to permit the onset of interactions between cancer cells and DRG within a drop of Matrigel (Fig. 1A). MiaPaCa2, a pancreatic adenocarcinoma line, showed a propensity to invade the Matrigel and track along neurites in a linear, unidirectional manner (Fig. 1A). The amount of PNI was quantified by measuring the area of cancer cells in contact with the DRG using MetaMorph software (Fig. 1A). In contrast, the head and neck squamous cell carcinoma cell line, QLL2, showed no ability to either invade the Matrigel drop or associate with neurites (Fig. 1B).

### GDNF-mediated cancer cell migration and DRG nerve invasion

We evaluated the ability of MiaPaCa2 to migrate towards soluble GDNF or DRG. Non-radiated MiaPaCa2 migrated towards GDNF, with a slightly stronger migratory behavior towards DRG (Fig. 1C). The amplified migratory effect by DRG suggests that nerve-secreted factors other than GDNF may play an additional role in promoting MiaPaCa2 chemotaxis towards nerves.

In the DRG-cancer co-culture assay in Matrigel, MiaPaCa2 exhibited an ability to invade the Matrigel and track along neurites towards the DRG. We found that the addition of anti-GDNF antibodies reduced the area of PNI by cancer cells as compared with controls treated with goat IgG (Fig. 1D). These findings suggest that PNI by MiaPaCa2 in this model is largely mediated through GDNF.

### Single fraction radiation doses and DRG viability

A single radiation dose of up to 20 Gy to DRG resulted in retained viability, with intact neurite growth and DRG morphology (data not shown). However, a single dose of 24 Gy resulted in death of DRG, with detachment from the Matrigel and loss of neurites by 4 days after radiation exposure (Fig. 2A). Therefore, a range of radiation doses up to 16 Gy was selected for use in the Boyden chamber and DRG-cancer co-culture *assays* (Fig. 1A) to ensure that observed effects were not confounded by impaired DRG viability.

### Radiation to DRG alone impairs PNI *in vitro*

There were no differences observed in the migration of MiaPaCa2 towards non-radiated or radiated DRG in migration assays performed over a 24 hour period (data not shown). However radiation of DRG used in the 8 day DRG-cancer co-culture assays resulted in a significant decrease in the area of invasion over an 8 day period. A range of single fraction doses of radiation were applied to the DRG, with no radiation of any cancer cells. Non-radiated DRG yielded a mean area of invasion exceeding 140,000 microns^2^. In sharp contrast, DRG radiated with a single dose of 4–16 Gy yielded mean areas of invasion less than 14,000 microns^2^ (Fig. 2B). No relationship between radiation dose and area of invasion was identified.

### Radiation of DRG decreases GDNF secretion

Conditioned media from radiated DRG was collected 8 days following radiation exposure and assayed for GDNF. Single-fraction doses from 4–16 Gy decreased measured GDNF (66 pg/ml) to undetectable levels as compared with non-radiated DRG (Fig. 2C). The assay limit of detection was 31 pg/mL.

The reduction of PNI resulting from radiation of DRG was partially rescued with the direct addition of GDNF to the DRG-Matrigel complex. Using the DRG-cancer co-culture assays, non-radiated DRG yielded a mean area of invasion of 170,000 microns^2^, while exposure of DRG to 4 Gy completely inhibited any measurable area of invasion at day 8. The addition of purified GDNF to the DRG-Matrigel complex resulted in a partial rescue, with a mean area of invasion of 73,000 microns^2^ (Fig. 2D). The lack of a full rescue of PNI with GDNF replacement may be related to the non-physiologic application of the GDNF in these experiments, an inability to recreate a continuous source of endogenous GDNF secretion by the DRG, and potential non-GDNF nerve secreted neurotrophic factors that might be diminished by radiation.

### Radiation of MiaPaCa2 cells impairs migration and PNI through compromised cellular viability

We evaluated if radiation may impair cancer cell migration and PNI through mechanisms other than cell death. All experiments with radiated cells were performed at least 24 hours after radiation to allow for sub-lethal damage repair. In colony formation assays, MiaPaCa2 plating efficiency for colony formation was 10%. Colony formation was susceptible to single-fraction radiation doses, showing diminishing surviving fractions up through 8 Gy (Fig. 3A) in a logarithmic fashion.

In migration assays, single fraction radiation of MiaPaCa2 inhibited cancer cell migration towards GDNF and DRG in a dose-dependent fashion (Fig. 3B). However, these observed differences correlated directly with surviving cell number in parallel plate experiments (Fig. 3B), suggesting that the impairment in migration was simply the result of RT-induced compromised cellular viability, rather than an impairment of chemotaxis. The assay was then repeated with starting cell numbers adjusted to achieve equivalent cell viability at 24 hours for all the radiation doses (data not shown), and differences in migration towards GDNF and DRG were eliminated.

Radiation exhibited similar effects in the DRG-cancer co-culture assay over an 8 day period. A single fraction of 2 Gy significantly decreased the mean area of invasion from 260,000 microns^2^ to 54,000 microns^2^, with dose response effects at higher doses (Fig. 3C). However, these decreases in areas of invasion correlated closely with cell viability as demonstrated by parallel plate cell counting over the identical time interval (Fig. 3C-D). These findings again suggest that compromised cellular viability is the predominant effect of radiation on the cancer cells in these models, rather than a more specific impairment of chemotactic function or perineural invasive ability.

### Radiation of the sciatic nerve alone diminishes PNI *in vivo*

To validate *in vitro* data suggesting that radiation to the nerve alone may impair PNI, we explored this concept *in vivo*. For selected animals, the entire course of a unilateral murine sciatic nerve was radiated, up to the spinal cord. One week later, non-radiated MiaPaCa2 cancer cells were injected into surgically exposed sciatic nerves (Fig. 4A). Mice tolerated single-fraction doses as high as 16 Gy to the sciatic nerve without any skin, soft tissue, muscular, or neurological toxicity. Sciatic nerve function remained normal following single dose radiation up to 16 Gy, and following the injection of cancer cells.

Mice with non-radiated sciatic nerves injected with non-radiated MiaPaCa2 cells develop progressive ipsilateral hind limb paralysis over 7 weeks, with extensive PNI by cancer cells by gross examination of the nerve (Fig. 4A). The normal murine sciatic nerve can be visualized *in vivo* by MRI and histologically (Fig. 4B) in a normal mouse. Mice with non-radiated sciatic nerves injected with non-radiated MiaPaCa2 cells underwent MRI at week 7 (Fig. 4B), which demonstrated anatomically thickened sciatic nerves that are consistent with PNI and histologically show extensive cancer infiltration along the nerve (Fig. 4B). In contrast, mice whose nerves were radiated with 4 Gy one week prior to injection with non-radiated MiaPaCa2 cells exhibited diminished sciatic nerve invasion by both MRI and histologic assessment (Fig. 4B).

For a functional assessment of sciatic nerves, mice were randomized to receive either 8 Gy or no radiation to the sciatic nerve 1 week prior to injection of non-radiated MiaPaCa2 cells. The sciatic neurological score (hind limb function) and sciatic nerve index (hindpaw width) were assessed. Mice receiving 8 Gy to the nerve showed significantly improved sciatic nerve function in comparison to mice with non-radiated nerves with impaired function and paralysis (Fig. 5B, C). Mice were sacrificed at week 7, and sciatic nerves were excised for gross (Fig. 5A) and histological assessment.

### Radiation of the sciatic nerve alone decreases GDNF production *in vivo*

To assess whether radiation alters the production of GDNF from sciatic nerves following radiation exposure, sciatic nerve lysates were collected 3 weeks after a single dose of 0 or 8 Gy and GDNF Elisa performed. A mean GDNF concentration of 130 pg/ml from non-radiated sciatic nerves was reduced to 65 pg/ml after a single dose of 8 Gy (Fig. 5D).

### Radiation to cancer cells alone diminishes PNI *in vivo*

Radiation to cancer inhibits PNI in our *in vitro* models predominantly through diminished cell viability, rather than impaired chemotaxis. To assess this concept *in vivo,* MiaPaCa2 received a single dose of 2 Gy, and 48 hours later were injected into non-radiated sciatic nerves. Compared with non-radiated MiaPaCa2 cells, low doses of radiation to the cancer resulted in diminished invasion on imaging and histopathological analysis at 7 weeks (Fig. 4B). Hind limb function was significantly improved in animals with radiated MiaPaCa2 cells as compared with controls (Fig. 6A, B, C, 5A). Tumor volume at 7 weeks was significantly lower for the radiated MiaPaCa2 cells, compared to non-radiated MiaPaCa2 control cells (Fig. 6D), suggesting that *in vivo* cell viability, proliferation, and tumor progression were significantly impaired by a single dose of 2 Gy.

### Expression of RET in human ACC cancer cells

Radiation is frequently administered to patients with ACC, a salivary gland cancer, following surgical resection because of its high propensity for PNI and local recurrence. We assessed whether patient ACC tumor specimens express RET, the cognate receptor for GDNF, using immunohistochemical staining. Hematoxylin-eosin staining frequently demonstrated well-formed ducts and nests of cells growing in hyalinized stroma in a mixed tubular and cribriform pattern (Fig. 7A). We identified mild to moderate levels of RET expression in all of the 18 human ACC tumor specimens assessed (Fig. 7B-D). The nerves and surrounding stromal tissues did not express RET. These findings suggest that our model of GDNF mediated RET signaling as a mechanism for chemotaxis and PNI may be clinically relevant for patients with ACC.

## Discussion

To date, there are only limited retrospective reports supporting the clinical application of radiation for the treatment of cancers exhibiting PNI. An understanding of how radiation may mechanistically treat PNI has been lacking. Radiation is traditionally thought to derive its main clinical benefit in this capacity by inducing the death of cancer cells present along an invaded nerve. In the current study, we elucidate a novel mechanism of how RT may inhibit PNI through the interruption of paracrine interactions between cancer cells and nerves. We demonstrate that a single dose of radiation may impair PNI through not only cancer cell death, but also independent effects on the nerve itself and the nerve's production of GDNF, a potent chemotactic factor.

Recent studies have pointed to the active role that the nerve microenvironment likely plays in the process of PNI [2], [21]. Rather than being a purely cancer induced event, as it was historically considered, PNI is now seen as the likely result of reciprocal interactions occurring between both cancer and nerve elements. Our group and others have recently demonstrated that PNI may be facilitated by neural secretion of GDNF, which phosphorylates the RET tyrosine kinase receptor when coupled with the GFRα receptor and triggers downstream signaling pathways that promote cancer cell migration and invasion [16], [18], [22]. Previous work from our laboratory demonstrated that DRG from mice deficient in GDNF (*gdnf +/−*) had significantly reduced *in vitro* invasion compared to wild type mice [18]. Further, we showed that the pharmacological inhibition of the GDNF-RET signaling pathway significantly suppressed nerve invasion and prevented paralysis in mice suggesting that GDNF was relevant *in vivo* as well [18].

We report here that radiation may impair PNI through the combination of independent effects on the cancer and the nerve. A single dose of radiation to the nerve significantly impaired PNI by non-radiated cancer cells, both *in vitro* in a DRG co-culture model and *in vivo* in a murine sciatic nerve model. Radiation effects on the nerve environment may therefore significantly contribute towards impairing PNI, even in the context of exposure to untreated cancer cells. Furthermore, we validated that PNI was dependent on GDNF *in vitro*, and that GDNF production by nerves was significantly impaired following radiation exposure in both *in vitro* and *in vivo* models. Other nerve-secreted neurotrophic and chemokine factors likely play contributing roles in PNI, with effects that might vary depending on specific cancer cell receptor expression [2], [21]. Further studies will be necessary to elucidate the potential effects of radiation on a nerve's production of other neurotrophic factors. Additionally, while not directly examined in our current study, nerve secreted ligands may potentially promote tumor cell survival. Therefore radiation induced suppression of such ligands may impair PNI through diminished tumor cell survival independent of the disruption in paracrine signaling and thereby contribute to some of our experimental findings. This potential radiation effect is worthy of future study.

As expected, radiation did induce potent direct effects on cancer cells that were independent of its effects on the nerve microenvironment. Experiments were conducted in which radiated cancer cells were studied in our models with normal, non-radiated nerves, and these consistently demonstrated a significant inhibition of migration and PNI. However, correlative cell viability assays performed in parallel with migration and PNI assays *in vitro* suggested that this effect was predominantly the result of compromised cellular viability, rather than a separate inhibition of cancer cell chemotactic or neural invasive abilities by the radiation. Similarly, *in vivo* tumor formation by radiated cancer cells was dramatically reduced, pointing to diminished cell viability and proliferation as the predominant mechanism of activity. Although the effects of radiation applied to both cancer and nerve together were not studied, we infer that combination therapy of both components is likely to be more effective in inhibiting PNI than of each component individually. In clinical practice, radiation to a patient with PNI would theoretically be delivered to both cancer cells and to the nerve microenvironment simultaneously.

The potential clinical relevance of these findings is underscored by our demonstration of the widespread expression of the GDNF receptor RET in human ACC specimens exhibiting PNI. High RET receptor expression has also been found in patients with pancreatic and prostate carcinomas, which also are known to exhibit frequent PNI behaviors [23], [24]. These findings suggest that the interruption of the GDNF-RET axis by radiation might be a clinically relevant strategy for treating PNI for a variety of cancers with a propensity for this phenotype.

This study has several limitations. First, potential non-GDNF neurotrophic factors (NGF, BDNF, artemin, CX3CL1, others) [21] participating in PNI were not explored in this study. Alternate cancer cell lines with PNI ability and varying cell surface receptor profiles were also not explored. Although alternate ligand-receptor interactions likely play a role in PNI mechanisms for other cell lines, these might be less applicable for MiaPaCa2 cells, where the GDNF-RET axis likely exerts the predominant effect [18]. Our deeper focus on MiaPaCa2 was intended to highlight the concept of radiation's ability to interrupt a paracrine mechanism of PNI. This novel idea may be extrapolated to other specific ligand-receptor systems in the future. A second limitation is that our studies necessarily separated, for mechanistic clarity, the independent effects of radiation on cancer and nerve. In clinical practice however, radiation is applied to both cancer and nerve simultaneously, and we suspect that combination effects might be enhanced. Other clinical parameters such as identifying optimal fractionation regimens also may be further evaluated in future studies.

Understanding the underlying mechanisms through which radiation may impair PNI is crucial towards improving our current treatment paradigms for patients with neurotrophic tumors. Current radiation planning for cancers with PNI involves designing radiation volumes that encompass the entire longitudinal course of an invaded cranial nerve back to its origin at the base of the skull, utilizing high doses intended to cause lethal damage to cancer cells. Such target volumes and associated doses carry the risk of toxicity due to the close proximity of adjacent critical structures such as the brain, optic nerves, optic chiasm, cochlea, and major salivary glands. A greater mechanistic understanding of PNI, and the effects of radiation on this process, might allow for optimization of radiation designs.

This study proposes a novel concept: a single low dose of radiation can impair PNI through not just cancer cell death, but also through an independent interruption of paracrine mechanisms underlying PNI. These findings suggest that radiation effects on the nerve microenvironment may derive additional therapeutic benefits not traditionally appreciated. The radiation doses considered necessary to inflict cancer cell death may not be necessary along all sites of the nerve and ganglia to disrupt neurotrophic signaling. It is possible that further investigation of this novel concept may ultimately lead to differential delivery of varying radiation doses along the course of the proximal nerve or ganglia, towards a goal of paracrine signal disruption rather than of cancer cell lethality. The ultimate goal of such innovative targeting strategies would be the enhancement of therapeutic control of PNI combined with the reduction in radiation induced toxicity.
